# Impact of an Active Nutrition Program on Weight Loss and Metabolic Health in Overweight and Obese Adults: A Randomized Controlled Trial

**DOI:** 10.7759/cureus.72545

**Published:** 2024-10-28

**Authors:** Michelle B Stockton, Jacquelyn Pence, Allyson Davis, Richard J Bloomer

**Affiliations:** 1 College of Health Sciences, University of Memphis, Memphis, USA

**Keywords:** diet, metabolic levels, nutrition, randomized controlled trial, weight loss

## Abstract

Background: The purpose of this study was to evaluate the impact of a 60-day low-calorie nutrition-rich diet plan compared to usual care on weight loss, physiological factors, markers of metabolic health, and perceived wellness.

Methods: Overweight or obese adults (N = 60) were randomly assigned to either the active nutrition group (n = 30) or the usual care group (n = 30). The active nutrition plan consisted of two meal replacement shakes, an electrolyte replacement drink, a metabolic support supplement, one whole food meal, and three to five servings of whole food snacks (e.g., vegetables and fruits), which totaled approximately 1200-1600 calories daily. Participants were evaluated at baseline and 30- and 60-day follow-ups for mean changes in anthropometric characteristics, biochemical variables, self-reported feelings of health and well-being, and dietary intake.

Results: Of the 60 participants, 49 were women and 11 were men, with a mean age of 38 ± 12 years. Adhering to the active nutrition program for 60 days resulted in a mean weight loss of approximately 6.5 pounds, with a 2-pound weight gain noted for subjects in the usual care group. Related to this, reductions were noted in BMI (p < 0.001), percent fat (p = 0.017), and waist (4 cm, p < 0.001) and hip (3 cm, p < 0.001) circumferences for the active nutrition group but not usual care. Clinically relevant changes were noted for reductions in blood pressure (5 mm Hg) and triglycerides (12 mg/dL) for the active nutrition group. From a subjective perspective, participants in the active nutrition group reported improvements in their self-esteem, motivation, endurance, physical appearance, and strength (p < 0.001). The above was achieved without participants reporting any significant increase in hunger during the intervention, despite the expected reduction in calories.

Conclusion: Adhering to a semi-structured diet plan that allows for one “regular” whole-food meal daily is well-tolerated and enhances weight loss, physiological factors, and perceived wellness. Follow-up studies with a balanced representation of biological men and women are warranted to determine the longer-term impact of this nutrition program on weight loss and markers of metabolic health.

## Introduction

Despite the multiple weight-loss interventions available, overweight status (defined by a body mass index (BMI) of 25-29.9 kg/m^2^) and obesity (defined as a BMI of ≥30 kg/m^2^) continue to be growing problems within the United States, with an estimated 42% of the population now categorized as obese and close to 75% categorized as either overweight or obese, according to the Centers for Disease Control and Prevention [1]. This public health problem has been strongly correlated with an increased risk of developing diseases, including type 2 diabetes, cardiovascular diseases, hypertension, and certain cancers [2]. While physical activity is an excellent method to combat weight gain, dietary intake is likely the most effective way to battle obesity, as decreasing the food that enters the body can almost always have a greater impact on creating a caloric deficit than increasing caloric expenditure through exercise [3-6].

In fact, studies have shown that for individuals who are overweight or moderately obese, diet is more effective at weight loss than exercise alone [3,7]. This is because the elimination of certain foods can easily lead to a 500-1000 calorie deficit per day, while yielding the same loss through exercise would require most individuals to exercise for one to two hours per day, something that has been shown to not be feasible for most adults [3,7]. Consistent with these findings, a meta-analysis of several weight-loss programs indicated that limiting the number of calories consumed combined with a focus on macronutrient composition were the main contributors to weight loss [8]. Moreover, obese individuals must restrict their caloric intake to a greater degree than individuals who have not been obese to lose weight [9].

The majority of physicians initially prescribe lifestyle modifications, such as changing dietary intake, to promote weight loss and help alleviate obesity and the associated chronic illnesses. A weight loss target of 5-15% is recommended for individuals who are overweight or obese with comorbidities [10-12]. Our prior work, inclusive of men and women who are overweight or obese, indicates that restriction of calories is met with weight loss but also favorable changes in health-related outcomes, such as fasting glucose and insulin, and improvements in antioxidant defenses [5,13-18]. Other investigators have noted similar findings, in addition to psychological and behavioral responses (i.e., decreased eating disorder symptoms, decreased depression, and increased quality of life) [19,20]. In most of our prior weight loss work, we have noted major changes within one to three weeks of beginning a lower-calorie nutrition plan.

Initial dieting success is well known to provide the motivation to continue because the significant weight loss during the first week of a diet plan provides a strong stimulus [12]. As such, many “dieters” find that an initial period of very low-calorie intake (e.g., 800-1000 calories per day) can greatly improve their longer-term weight loss success, likely by providing motivation (due to the significant and immediate weight loss results) that allows them to continue with their weight loss pursuit [21]. A loss of 3-5 pounds within the initial seven days of a very low-calorie diet is not uncommon and we have recently reported a decrease in 4.5 pounds in a sample of 26 men and women adhering to the USANA Active Nutrition Jumpstart nutrition program [22]. This one-week nutrition plan consists of meal replacement shakes and supportive supplements that can help facilitate weight loss and provide adequate nutritional support (macro- and micro-nutrients) during a low-calorie feeding period. Moreover, meal replacements are preferred by many busy individuals, as these products are nutrient-dense (i.e., high nutrient content, allowing for a lower caloric intake while continuing to provide adequate nutrients to maintain health), while proving to be very convenient [23]. It is clear that when convenient options are provided, dietary adherence is improved, resulting in greater weight loss success over time [23-25].

The 60-day Active Nutrition program was designed to evaluate the efficacy of a weight loss program in a sample of overweight and obese men and women. The program consisted of two meal replacement shakes per day (typically consumed for breakfast and lunch), in addition to one sensible whole-food meal of ~400-700 calories. In addition, four to five servings of fruit and/or vegetables were consumed daily, yielding a daily intake of approximately 1200-1600 calories. A daily electrolyte replacement drink was consumed to prevent dehydration. A supplement to boost metabolism was also consumed three times daily at each meal. The study did not only evaluate weight loss and changes in body shape but also assessed the type of weight loss (fat vs. lean mass) and other health measures affected by obesity including lipids, glucose, resting heart rate and blood pressure, and perceived wellness. We hypothesized that the Active Nutrition program would result in greater weight loss, as well as improvements in bloodborne markers of health, as compared to usual care (i.e., no change in diet).

This article was previously presented as a meeting abstract at the 2024 ACSM Annual Scientific Meeting on May 29, 2024.

## Materials and methods

Study design

This study was a two-group randomized controlled trial with assessments completed on-site at the university research facility. Participants were randomly assigned to either the 60-day Active Nutrition group or a usual care group (control) with consideration to balance for BMI, sex, and age within groups. The primary outcomes were anthropometric measures, including weight, BMI, waist and hip circumference, percent fat, and lean body mass taken at baseline, 30 days, and 60 days. Secondary outcomes included markers of metabolic health (insulin, cholesterol, high-density lipoprotein (HDL), triglycerides, glucose, non-HDL-cholesterol (nHDLc), total cholesterol/HDL (TC/H), low-density lipoprotein (LDL), and very-low-density lipoprotein (VLDL)) and participant perceptions of wellness at baseline, 30 days, and 60 days. The study was approved by the University of Memphis Institutional Review Board for Human Subject Research (PRO-FY2022-181). This trial was registered at www.ClinicalTrials.gov (Identifier: NCT06249698). USANA provided a sponsored grant to the University of Memphis to support the independent research. The supplements and nutrient shakes used in the study were also supplied by USANA. All potential conflicts of interest were transparently disclosed to the journal for review. No author has a financial affiliation with USANA.

Study participants

Participants included men and women aged 18-60 years of age who had a BMI of 28-39.9 kg/m^2^. To be eligible, individuals needed to be able to fast overnight (>10 hours) and be willing to maintain existing exercise levels through the duration of study participation. Individuals could not be diagnosed with type 1 or type 2 diabetes or liver disease, could not use tobacco products, reported no adversity to fiber or protein supplements, were not currently taking weight loss dietary supplements or adhering to any weight loss plan in the month prior to and during participation in the study, were not currently taking a multivitamin or probiotic (or if they were not recommended by a doctor, be willing to stop for four weeks prior to the study). Study exclusionary criteria included those who are currently pregnant, lactating, or intending to become pregnant and have an active infection or illness of any kind. In addition, 24 hours prior to testing participants could not have any alcohol-containing or caffeine-containing beverages and could not have any strenuous exercise.

Recruitment and screening

Participants were recruited via social media advertisements, email communications, and word of mouth. Those interested contacted the study recruiter and completed a brief telephone screening to determine whether they met the basic study requirements. Once initially approved, potential participants were scheduled for their initial screening visit.

During the initial screening laboratory visit, participants completed the informed consent form and health history questionnaire. The health history form was reviewed by the investigators for any contraindicators. Subjects’ heart rate and blood pressure, height, weight, waist, and hip circumference were measured. Biological women were provided with a urine pregnancy test kit at the screening visit and each study visit. They were escorted to a private restroom within the lab and asked to perform the test to confirm that they were not pregnant.

Upon completion of the initial screening measures, eligible subjects were scheduled for testing. Subjects were assigned a subject number for privacy protection and scheduled for their initial testing visit. Pre-menopausal women began the study during the first 5 days of their menstrual cycle to control for circulating hormones that are known to fluctuate during the cycle and potentially influence outcome measures.

Interventions

All subjects underwent an intervention to determine changes in pre- and post-intervention outcomes. The interventions consisted of following either usual care (adherence to the usual diet for the entire study period) or the Active Nutrition plan for 60 days.

Participants assigned to the usual care control group were instructed to adhere to their usual dietary practices, with regard to food and drink type and quantity. Participants in the Active Nutrition group consumed the Nutrimeal Active™ replacement shake (whey protein, fructose, sunflower oil, maltodextrin, fructooligosaccharide, natural flavors, vitamin and mineral blend (magnesium citrate, tricalcium phosphate, dipotassium phosphate, zinc gluconate, ascorbic acid, niacinamide (niacin), manganese citrate, copper gluconate, vitamin E acetate, biotin, vitamin a acetate, sodium selenite, potassium iodide, calcium pantothenate, molybdenum citrate, chromium chloride, vitamin D3, folic acid, riboflavin, pyridoxine hydrochloride, and thiamine hydrochloride vitamin B12), modified tapioca starch, sunflower lecithin, sugarcane fiber, organic agave inulin, flaxseed, sodium carboymethylcellulose, modified food starch, sea salt, xantham gum, blueberry powder, apple powder, raspberry powder, monk fruit extrac, stevia Reb, and ferrous fumarate) at breakfast and either lunch or dinner (240 calories, 9 g fat, 24 g carbohydrate, 8 g fiber, 20 g protein per 10-12 oz shake), an Electrolyte Replacement Drink Mix™ (calcium citrate, magnesium, sodium citrate, potassium citrate, cane sugar, maltodextrin, sea salt, microcrystalline cellulose, gum acacia, vegetable color, natural watermelon flavor, citric acid, and steviol glucoside) once a day to help with dehydration due to increased protein and decreased carbohydrate intake, one Metabolism+™ Weight Support Supplement (green tea leaf extract, platycodon root extract, USANA citrus bioflavonoid complex (naringin and hesperidin), microstrystalline cellulose, vegetable fatty acid, croscarmellose sodium, modified cellulose, dextrin, silicon dioxide, natural grape flavor, dextrose, soy lecithin, stevia, sodium carboymethylcellulose, and sodium citrate) taken with each meal replacement shake and whole food meal (total of three servings per day), one whole food meal at either dinner or lunch, participants’ choice (400-700 calories), whole foods snacks that consist of primarily fruits and vegetables (aiming for three to five total servings per day; ~350 calories). For items within the Active Nutrition plan, the contract manufacturer (USANA USA, Salt Lake City, Utah) produced all products in accordance with Good Manufacturing Practices.

Considering the above, the participants should have ingested approximately 1200-1600 calories daily, which is typical of most weight loss diet programs, but much more than a standard “very low-calorie diet” of 800 calories per day, which is often recommended for significant weight loss [26-28].

Test visit procedures

The participants reported to the lab following an overnight fast (>10 hours) on three days (day 1, day 30, and day 60). All assessments were taken by research staff not involved in the intervention, who were trained using standardized protocols. The following outcomes were measured: body weight, waist and hip circumference, BMI, body composition (via DXA Hologic Horizon A), resting heart rate, resting blood pressure, blood lipids (Piccolo Xpress, Abbott), glucose (Contour, Bayer), insulin (Insulin ELISA IN374S, Calbiotech), and participants’ perceived wellness variables (self-esteem, energy level, motivation, irritability, focus, hunger, endurance, life satisfaction, physical appearance, and strength) via questionnaire using the standardized 100 mm Adaptive Visual Analog Scales.

Upon arrival at the lab, the participants were asked to void their bladder and bowels. Females self-administered a urine pregnancy test. They rested for 10 minutes and had their heart rate and blood pressure taken. Body weight was measured wearing only underwear and a gown, circumference measures were taken, as well as a blood sample. Single venipunctures were used to collect blood samples from subjects during testing visits. Approximately 5 mL of blood was taken at each draw, processed, and analyzed for lipids, glucose, and insulin. A whole-body dual-energy X-ray absorptiometry (DXA) scan was conducted for the measurement of body composition (Horizon A scanner; Hologic, Marlborough, MA). Subjects completed a subjective 10-item assessment rating feelings of wellness on a scale from 1 to 10. To coincide with the waist and hip measurements, if the participants consented, digital photos of the midsection and hip area only were taken from the front, side, and back and stored on a password-protected computer (photos not included in this manuscript). The laboratory visit lasted approximately one hour, and subsequent visits were scheduled at that time. At the end of the 60-day visit, the participants completed the protocol and were debriefed.

The participants followed their usual activity patterns over the course of the study period but were asked to refrain from strenuous activity for the 24 hours preceding each lab test day. No alcohol should have been consumed during the 24 hours prior to each lab test day. Diets should have consisted only of the items indicated above (i.e., supplements, fruits and vegetables, and whole food meals), as well as non-calorie-containing beverages. Dietary records were maintained for the five days prior to each test day. Participants recorded their detailed dietary intake on food logs for five days prior to each test visit. This included all food and drink consumed. Dietary data were analyzed for total kcal and macro- and micro-nutrient composition using Food Processor Pro software (Esha Research; Salem, OR).

Data analysis

The assumptions of outliers and normal distribution of the data were inspected by using box plots, normal probability plots, and the Shapiro-Wilk test of normality. Descriptive statistics were used to outline baseline characteristics (means, standard deviations, frequencies, and percentages). Mean changes in anthropometric variables, body composition, biochemical variables, self-reported feelings, and nutrients at baseline and days 30 and 60 were assessed using two-way mixed analyses of variance. All analyses were performed using the IBM SPSS Statistics for Windows (version 28; IBM Corp., Armonk, NY). All statistical tests were two-tailed with the significance level represented by p < 0.05.

## Results

Study population

**Figure 1:**
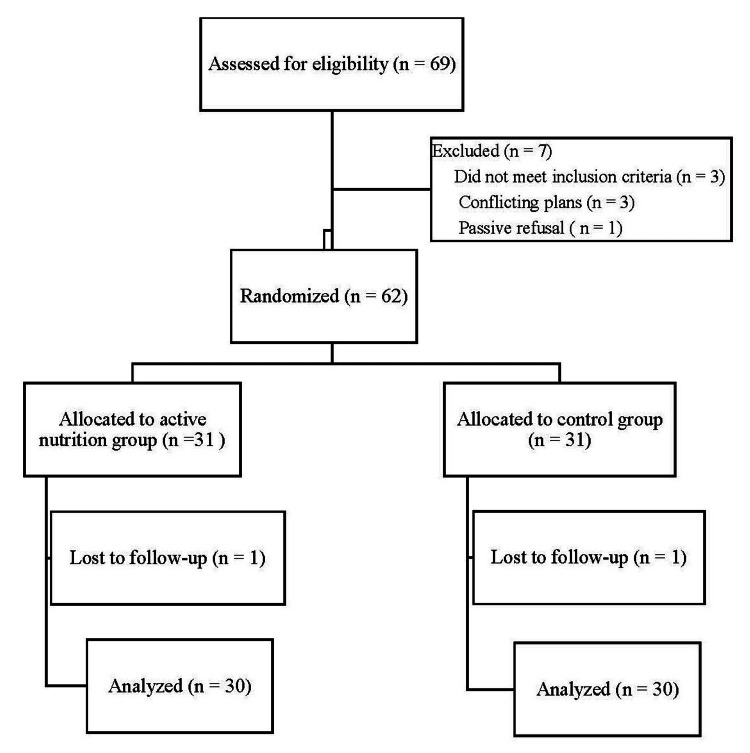
Enrollment and retention

Power analysis for a repeated-measures ANOVA with two groups and three time points was conducted in G*Power version 3.1.9.4 (Universität Düsseldorf, Germany) to determine a sufficient sample size using an alpha of 0.05, a power of 0.80, and a large effect size (f2 = 0.40). Based on the aforementioned assumptions, the desired total sample size was 46 (23 per group). The sample size was set at 60 to account for attrition. A total of 60 participants completed the study. Nine participants provided informed consent but failed to complete the study due to various reasons, as described in Figure 1.

Of the 60 subjects, 49 were women and 11 were men, with a mean age of 38 ± 12 years. There were no adverse events reported related to any of the observed outcomes, although one subject reported a hospitalization for a pre-existing condition (unrelated to study participation or test product). This participant remained in the study and was able to complete the study protocol. See Table 1 for full details.

**Table 1:** Baseline subject characteristics by condition (mean (SD) or n (%))

Characteristics	Control (n = 30)	Active (n = 30)	Total (n = 60)
Age (yrs)	33.7 (10.1)	42.8 (11.5)	38.3 (11.7)
Gender, n (%)			
Male	5 (16.1)	6 (20.0)	11 (18.3)
Female	25 (83.9)	24 (80.0)	49 (81.7)
Height (cm)	166.8 (5.9)	165.6 (10.9)	166.2 (8.7)
Weight (kg)	92.0 (8.8)	91.8 (15.7)	91.9 (12.6)
BMI (kg/m^2^)	33.1 (2.9)	33.3 (3.5)	33.2 (3.2)
Waist (cm)	97.3 (8.9)	99.1 (12.8)	98.2 (11.0)
Hip (cm)	114.3 (7.7)	116.3 (7.2)	115.3 (7.5)
Systolic Blood Pressure (mm Hg)	117.3 (8.3)	120.6 (14.8)	118.9 (12.0)
Diastolic Blood Pressure (mm Hg)	78.8 (6.9)	80.0 (9.7)	79.4 (8.3)
Heart Rate (bpm)	76.9 (9.2)	71.8 (9.0)	74.4 (9.4)

Outcomes

Anthropometric Characteristics

The two-way mixed ANOVAs indicated that there were statistically significant interactions between the treatment groups and time on weight (F(1.481, 85.899) = 43.00, p < 0.001, partial η2 = 0.426); BMI (F(1.543, 89.489) = 44.34, p < 0.001, partial η2 = 0.433); waist circumference (F(1.565, 90.792) = 49.49, p < 0.001, partial η2 = 0.460); hip circumference (F(1.478, 85.742) = 40.59, p < 0.001, partial η2 = 0.412), fat mass (F(1.668, 96.728) = 21.62, p < 0.001, partial η2 = 0.272); lean mass (F(2, 116) = 18.98, p < 0.001, partial η2 = 0.247); percent fat (F(2, 116) = 3.82, p = 0.025, partial η2 = 0.062); and percent lean (F(2, 116) = 3.82, p = 0.025, partial η2 = 0.062). There were no significant differences found for bone mass. See Table 2 for details.

**Table 2:** Anthropometric characteristics of participants during the pre-treatment, visit 1 (one month), and visit 2 (two months) SBP: systolic blood pressure, DBP: diastolic blood pressure, HR: heart rate

Outcome	Control (Mean ± SD)	Active (Mean ± SD)	p-value (two-way mixed ANOVA)
Weight (kg)			<0.001
Pre	91.46 + 8.69	91.29 + 15.22	
Visit 1	92.05 + 8.96	89.01 + 14.73	
Visit 2	92.43 + 9.51	88.28 + 14.75	
BMI			<0.001
Pre	32.89 + 2.86	33.14 + 3.39	
Visit 1	33.10 + 2.95	32.34 + 3.53	
Visit 2	33.23 + 3.08	32.07 + 3.56	
Waist (cm)			<0.001
Pre	96.83 + 8.94	98.68 + 12.76	
Visit 1	96.73 + 9.04	95.90 + 12.69	
Visit 2	96.22 + 9.21	94.55 + 12.44	
Hip (cm)			<0.001
Pre	114.20 + 7.80	116.13 + 7.10	
Visit 1	114.18 + 7.54	114.25 + 7.24	
Visit 2	114.23 + 7.67	113.20 + 7.21	
Fat (g)			<0.001
Pre	32414.20 + 8533.83	33551.43 + 8303.99	
Visit 1	32902.67 + 8366.22	32481.60 + 8457.99	
Visit 2	33038.17 + 8625.57	32057.40 + 8865.87	
Lean (g)			<0.001
Pre	57096.71 + 8193.66	56171.55 + 13549.60	
Visit 1	57376.38 + 8439.00	54986.36 + 12911.24	
Visit 2	57869.53 + 8741.61	54592.80 + 12947.08	
Bone (g)			.054
Pre	2397.09 + 357.58	2251.55 + 445.90	
Visit 1	2393.02 + 359.30	2277.67 + 476.06	
Visit 2	2400.64 + 362.38	2264.40 + 468.03	
% Fat			0.025
Pre	35.14 + 8.02	36.76 + 7.46	
Visit 1	35.42 + 7.92	36.39 + 7.63	
Visit 2	35.31 + 8.06	36.22 + 7.88	
% Lean			.025
Pre	64.86 + 8.02	63.24 + 7.46	
Visit 1	64.58 + 7.92	63.61 + 7.63	
Visit 2	64.69 + 8.06	63.78 + 7.88	
SBP (mmHg)			.037
Pre	113.60 + 7.36	118.33 + 12.25	
Visit 1	113.03 + 7.76	112.77 + 11.54	
Visit 2	111.57 + 8.06	112.70 + 11.49	
DBP (mmHg)			.006
Pre	77.27 + 7.17	79.17 + 9.08	
Visit 1	78.33 + 6.59	75.27 + 9.49	
Visit 2	76.13 + 8.32	75.00 + 9.42	
HR (bpm)			.369
Pre	75.83 + 9.57	73.20 + 9.36	
Visit 1	75.23 + 9.12	69.83 + 11.85	
Visit 2	74.43 + 8.46	71.03 + 11.29	

There were several simple main effects for time for the Active Nutrition group only. There were no significant pairwise comparisons for the control group. The Active Nutrition group was statistically significantly different across the time points on weight (p < 0.001) with pairwise comparisons being significantly different for weight between the pre-visit vs. visit 1 (Mpre = 91.29, Mv1 = 89.01; p < 0.001); pre-visit vs. visit 2 (Mpre = 91.29, Mv2 = 88.28; p < 0.001); and visit 1 vs. visit 2 (Mv1 = 89.01, Mv2 = 88.28; p = 0.006). The Active Nutrition group was statistically significantly different across the time points on BMI (p < 0.001) with pairwise comparisons being significantly different for BMI between the pre-visit vs. visit 1 (Mpre = 33.14, Mv1 = 32.34; p < 0.001); pre-visit vs. visit 2 (Mpre = 33.14, Mv2 = 32.07; p < 0.001); visit 1 vs. visit 2 (Mv1 = 32.34, Mv2 = 32.07; p = 0.006). The Active Nutrition group was statistically significantly different across the time points on waist circumference (p < 0.001) with pairwise comparisons being significantly different for waist circumference between the pre-visit vs. visit 1 (Mpre = 98.68, Mv1 = 95.90; p < 0.001); pre-visit vs. visit 2 (Mpre = 98.68, Mv2 = 94.55; p < 0.001); visit 1 vs. visit 2 (Mv1 = 95.90, Mv2 = 94.55; p < 0.001). The Active Nutrition group was statistically significantly different across the time points on the hip circumference (p < 0.001) with pairwise comparisons being significantly different for the hip circumference between the pre-visit vs. visit 1 (Mpre = 116.13, Mv1 = 114.25; p < 0.001); pre-visit vs. visit 2 (Mpre = 116.13, Mv2 = 113.20; p < 0.001); visit 1 vs. visit 2 (Mv1 = 114.25, Mv2 = 113.20; p < 0.001).

The Active Nutrition group was statistically significantly different across the time points on fat mass (p < 0.001) with significant pairwise comparisons between the pre-visit vs. visit 1 (Mpre = 33551.43, Mv1 = 32481.60; p < 0.001) and pre-visit vs. visit 2 (Mpre = 33551.43, Mv2 = 32057.40; p < 0.001). However, visit 1 was not significantly different than visit 2 (Mv1 = 32481.60, Mv2 = 32057.40; p = 0.089). The Active Nutrition group was statistically significantly different across the time points on lean mass (p < 0.001), with significant pairwise comparisons between the pre-visit vs. visit 1 (Mpre = 56171.55, Mv1 = 54986.36; p < 0.001); pre-visit vs. visit 2 (Mpre = 56171.55, Mv2 = 54592.80; p < 0.001). However, visit 1 was not significantly different than visit 2 (Mv1 = 54986.36, Mv2 = 54592.80; p = 0.197).

The Active Nutrition group was statistically significantly different across the time points on percent fat (p = 0.017) with a significant pairwise comparison between the pre-visit and visit 2 (Mpre = 36.76, Mv2 = 36.21; p = 0.037). However, the following were not statistically significantly different: (pre-visit and visit 1 (Mpre = 36.76, Mv1 = 36.39; p = 0.185); visit 1 and visit 2 (Mv1 = 36.39, Mv2 = 36.21; p = 0.901). The Active Nutrition group was statistically significantly different across the time points on percent lean (p = 0.017) with a significant pairwise comparison between the pre-visit and visit 2 (Mpre = 63.24, Mv2 = 63.78; p = 0.037). However, the following were not statistically significantly different: (pre-visit and visit 1 (Mpre = 63.24, Mv1 = 63.61; p = 0.185); visit 1 and visit 2 (Mv1 = 63.61, Mv2 = 63.78; p = 0.901)).

The Active Nutrition group was statistically significantly different across the time points on SBP (p < 0.001), with significant pairwise comparisons between the pre-visit and visit 1 (Mpre = 118.33, Mv1 = 112.77; p = 0.002) and pre-visit and visit 2 (Mpre = 118.33, Mv2 = 112.77; p = 0.011). However, there was not a statistically significant difference between visit 1 and visit 2 (Mv1 = 112.77, Mv2 = 112.77; p = 1.00). The Active Nutrition group was statistically significantly different across the time points on DBP (p < 0.001) with significant pairwise comparisons between the pre-visit and visit 1 (Mpre = 79.17, Mv1 = 75.27; p = 0.003) and pre-visit and visit 2 (Mpre = 79.17, Mv2 = 75.00; p = 0.003). However, there was not a statistically significant difference between visit 1 and visit 2 (Mv1 = 75.27, Mv2 = 75.00; p = 1.00).

Hemodynamics and Blood-Borne Values

The two-way mixed ANOVAs indicated that there were statistically significant interactions between the treatment groups and time on triglycerides (F(1.549, 89.859) = 4.47, p = 0.022, partial η2 = 0.071) and on VLDL (F(1.533, 88.920) = 4.81, p = 0.017, partial η2 = 0.077). There were no significant differences found for insulin, cholesterol, HDL, glucose, nHDLc, TC/H, and LDL. See Table 3 for details.

**Table 3:** Hemodynamics and blood-borne values of participants during the pre-treatment, visit 1 (one month), and visit 2 (two months) Chol: cholesterol, HDL: high-density lipoprotein, Trig: triglycerides, GLU: glucose, nHDLc: non-HDL-cholesterol, TC/H: total cholesterol/HDL ratio, LDL: low-density lipoprotein, VLDL: very-low-density lipoprotein

Outcome	Control (Mean ± SD)	Active (Mean ± SD)	p-value (two-way mixed ANOVA)
Insulin (µU/mL)			.451
Pre	26.76 + 13.60	21.84 + 15.05	
Visit 1	26.32 + 14.58	21.72 + 24.72	
Visit 2	28.68 + 17.00	18.64 + 11.65	
Chol (mg/dL)			.062
Pre	175.10 + 31.71	181.27 + 31.71	
Visit 1	173.63 + 34.14	168.87 + 26.90	
Visit 2	174.80 + 36.55	174.27 + 28.56	
HDL (mg/dL)			.126
Pre	52.13 + 9.15	57.53 + 10.87	
Visit 1	50.93 + 11.34	52.47 + 9.51	
Visit 2	51.37 + 10.27	55.50 + 10.16	
Trig (mg/dL)			.022
Pre	98.33 + 40.52	104.63 + 38.47	
Visit 1	95.43 + 41.22	98.77 + 37.88	
Visit 2	109.07 + 47.95	92.77 + 35.98	
GLU (mg/dL)			.422
Pre	86.57 + 7.76	88.07 + 10.19	
Visit 1	83.23 + 8.88	86.03 + 8.87	
Visit 2	85.43 + 8.97	85.57 + 11.81	
nHDLc (mg/dL)			.169
Pre	122.90 + 30.75	123.63 + 28.98	
Visit 1	122.87 + 30.23	116.37 + 25.42	
Visit 2	123.50 + 35.32	118.87 + 26.45	
TC/H			.566
Pre	3.44 + 0.76	3.23 + 0.73	
Visit 1	3.53 + 0.85	3.30 + 0.75	
Visit 2	3.52 + 0.92	3.21 + 0.65	
LDL (mg/dL)			.190
Pre	103.07 + 27.87	102.73 + 27.34	
Visit 1	103.63 + 24.95	96.73 + 23.34	
Visit 2	101.73 + 29.89	100.20 + 26.08	
VLDL (mg/dL)			.017
Pre	19.60 + 8.14	20.93 + 7.63	
Visit 1	19.10 + 8.28	19.83 + 7.58	
Visit 2	21.83 + 9.60	18.50 + 7.24	

Although there were no significant differences found for insulin, upon inspection of the data, a one-way ANOVA was conducted and indicated that there was a statistically significant difference in mean insulin levels at visit 2 between the active nutrition group (M = 18.64, SD = 11.65) and the control group (M = 28.68, SD = 17.00), F (1, 58) = 7.12, p = 0.010. The previsit and visit 1 time points were not significantly different between the two groups (p = 0.189, p = 0.383, respectively).

The results for simple main effects for time indicated that the Active Nutrition group was not statistically significantly different across the time points on triglycerides (p = 0.209) although the means are trending in the right direction (Mpre = 104.63, Mv1 = 98.77, Mv2 = 92.77). However, the control group was statistically significantly different across the time points on triglycerides (p = 0.030), but not in the desired direction with a significant pairwise comparison between visit 1 and visit 2 (Mv1 = 95.43, Mv2 = 109.07; p = 0.018).

Simple main effects for time indicated that the Active Nutrition group was not statistically significantly different across the time points on VLDL (p = 0.195). Simple main effects for time indicated that the control group was statistically significantly different across the time points on VLDL (p = 0.026). There was a significant pairwise comparison between visit 1 and visit 2 (Mv1 = 19.10, Mv2 = 21.83; p = 0.015). However, there was not a statistically significant difference between the pre-Visit and visit 1 (Mpre = 19.60, Mv1 = 19.10; p = 1.00) nor between the pre-visit and visit 2 (Mpre = 19.60, Mv2 = 21.83; p = 0.225).

Perceived Wellness

The two-way mixed ANOVAs indicated that there were statistically significant interactions between the treatment groups and time on self-esteem (F(1.599, 92.722) = 8.57, p < 0.001, partial η2 = 0.129); motivation (F(2, 116) = 4.56, p = 0.012, partial η2 = 0.073); endurance (F(2, 116) = 7.08, p = 0.001, partial η2 = 0.109); physical appearance (F(1.790, 103.792) = 4.22, p = 0.021, partial η2 = 0.068); and strength (F(1.731, 100.383) = 4.43, p = 0.019, partial η2 = 0.071). There were no significant differences found for energy, irritability, focus, hunger, and life satisfaction. See Table 4 for details.

**Table 4:** Subjective feelings of the participants during the pre-treatment, visit 1 (one month), and visit 2 (two months)

Outcome	Control (Mean ± SD)	Active (Mean ± SD)	p-value (two-way mixed ANOVA)
Self-esteem			<0.001
Pre	6.69 + 1.98	6.28 + 1.77	
Visit 1	6.61 + 1.93	7.09 + 1.32	
Visit 2	6.83 + 1.70	7.59 + 1.21	
Energy			.110
Pre	5.45 + 1.75	5.40 + 1.70	
Visit 1	6.05 + 1.89	6.41 + 1.59	
Visit 2	6.00 + 1.88	6.86 + 1.47	
Motivation			0.012
Pre	5.73 + 1.79	5.34 + 1.85	
Visit 1	5.87 + 1.96	6.70 + 1.52	
Visit 2	6.46 + 1.40	7.07 + 1.66	
Irritability			0.402
Pre	4.33 + 2.10	4.39 + 2.06	
Visit 1	4.49 + 1.76	4.03 + 2.25	
Visit 2	3.85 + 1.84	4.09 + 1.88	
Focus			0.262
Pre	5.86 + 1.41	5.75 + 1.70	
Visit 1	5.55 + 1.45	6.17 + 1.52	
Visit 2	6.28 + 1.59	6.43 + 1.35	
Hunger			0.573
Pre	5.18 + 1.59	4.53 + 2.02	
Visit 1	4.90 + 1.76	4.75 + 1.96	
Visit 2	5.25 + 1.58	4.57 + 2.17	
Endurance			0.001
Pre	5.96 + 1.77	5.08 + 1.95	
Visit 1	5.86 + 1.74	6.28 + 1.71	
Visit 2	5.95 + 1.83	6.79 + 1.39	
Life Satisfaction			0.195
Pre	6.91 + 1.47	6.59 + 1.56	
Visit 1	7.07 + 1.44	7.15 + 1.70	
Visit 2	7.22 + 1.53	7.46 + 1.52	
Physical Appearance			0.021
Pre	5.18 + 2.00	4.90 + 1.95	
Visit 1	5.46 + 1.96	6.14 + 1.87	
Visit 2	6.03 + 1.95	6.52 + 1.81	
Strength			0.019
Pre	5.81 + 1.37	4.98 + 1.86	
Visit 1	6.32 + 1.48	6.07 + 1.42	
Visit 2	6.38 + 1.52	6.65 + 1.42	

There was a significant finding between the groups for self-esteem at visit 2 only, F(1, 58) = 4.02, p = 0.050, partial η2 = 0.065, with the Active Nutrition group (M = 7.59, SE = 0.270) having statistically significant higher self-esteem than the control group (M = 6.825, SE =0.270).

There were several simple main effects for time for the Active Nutrition group only. There were no significant pairwise comparisons for the control group. The Active Nutrition group was statistically significantly different across the time points on self-esteem (p < 0.001) with significant pairwise comparisons between the pre-visit and visit 1 (Mpre = 6.28, Mv1 = 7.09; p < 0.001); the pre-visit and visit 2 (Mpre = 6.28, Mv2 = 7.09; p < 0.001); and visit 1 and visit 2 (Mv1 = 7.09, Mv2 = 7.59; p = 0.002). The Active Nutrition group was statistically significantly different across the time points on motivation (p < 0.001) with significant pairwise comparisons between the pre-visit and visit 1 (Mpre = 5.34, Mv1 = 6.70; p = 0.002); the pre-visit and visit 2 (Mpre = 5.34, Mv2 = 7.07; p < 0.001). There was not a significant pairwise comparison for motivation between visit 1 and visit 2 (Mv1 = 6.70, Mv2 = 7.07; p = 0.495). The Active Nutrition group was statistically significantly different across the time points on endurance (p < 0.001) with significant pairwise comparisons for endurance between the pre-visit and visit 1 (Mpre = 5.08, Mv1 = 6.28; p = 0.009); the pre-visit and visit 2 (Mpre = 5.08, Mv2 = 6.79; p < 0.001). There was not a significant pairwise comparison for endurance between visit 1 and visit 2 (Mv1 = 6.28, Mv2 = 6.79; p = 0.315). The Active Nutrition group was statistically significantly different across the time points on physical appearance (p < 0.001) with significant pairwise comparisons for physical appearance between the pre-visit and visit 1 (Mpre = 4.90, Mv1 = 6.14; p < 0.001); the pre-visit and visit 2 (Mpre = 4.90, Mv2 = 6.52; p < 0.001). There was not a significant pairwise comparison for physical appearance between visit 1 and visit 2 (Mv1 = 6.14, Mv2 = 6.52; p = 0.294). The Active Nutrition group was statistically significantly different across the time points on strength (p < 0.001) with significant pairwise comparisons for strength between the pre-visit and visit 1 (Mpre = 4.98, Mv1 = 6.07; p = 0.005); pre-visit and visit 2 (Mpre = 4.98, Mv2 = 6.65; p < 0.001); visit 1 and visit 2 (Mv1 = 6.07, Mv2 = 6.65; p = 0.030).

Dietary Intake

The two-way mixed ANOVAs indicated that there were statistically significant interactions between the treatment groups and time and in the expected direction for calories (p < 0.001), carbohydrates (p = 0.001), total fiber (p < 0.001), fat (p < 0.001), cholesterol (p = 0.002), vitamin C (p = 0.037), vitamin D (p < 0.001), and calcium (p < 0.001), but no differences for protein and sugar (p > 0.05). See Table 5 for details.

**Table 5:** Dietary intake of the participants during the pre-treatment, visit 1 (one month), and visit 2 (two months)

Outcome	Control (Mean ± SD)	Active (Mean ± SD)	p-value (two-way mixed ANOVA)
Calories (kcal)			<0.001
Pre	1657.46 + 476.60	1690.25 + 583.08	
Visit 1	1621.59 + 461.50	1236.95 + 215.17	
Visit 2	1776.28 + 464.98	1230.10 + 272.41	
Protein (g)			0.082
Pre	72.02 + 25.77	77.43 + 30.00	
Visit 1	72.16 + 24.64	76.01 + 19.53	
Visit 2	77.13 + 24.31	73.08 + 18.02	
Carbohydrates (g)			0.001
Pre	185.27 + 64.36	181.07 + 64.60	
Visit 1	173.86 + 57.92	139.82 + 28.30	
Visit 2	188.22 + 52.99	136.29 + 30.92	
Total Fiber (g)			<0.001
Pre	13.79 + 7.07	13.85 + 4.85	
Visit 1	13.24 + 5.45	25.45 + 4.45	
Visit 2	14.00 + 4.75	24.15 + 4.81	
Sugar (g)			0.925
Pre	62.74 + 26.07	62.12 + 35.44	
Visit 1	57.40 + 29.11	60.14 + 14.12	
Visit 2	57.64 + 20.42	57.89 + 13.85	
Fat (g)			<0.001
Pre	67.97 + 20.60	69.71 + 25.32	
Visit 1	69.43 + 22.29	44.18 + 12.01	
Visit 2	78.37 + 23.46	45.54 + 13.87	
Cholesterol (mg)			0.002
Pre	288.09 + 176.44	256.78 + 149.95	
Visit 1	280.37 + 121.56	176.45 + 173.34	
Visit 2	323.04 + 179.22	144.62 + 76.37	
Vitamin C (mg)			0.037
Pre	48.22 + 47.81	60.84 + 63.93	
Visit 1	78.14 + 195.30	81.31 + 66.32	
Visit 2	77.45 + 190.11	86.00 + 73.73	
Vitamin D (mcg)			<0.001
Pre	2.39 + 2.10	2.31 + 2.25	
Visit 1	2.04 + 1.73	11.47 + 1.93	
Visit 2	3.03 + 2.36	10.68 + 2.31	
Calcium (mg)			<0.001
Pre	487.06 + 196.53	635.36 + 312.00	
Visit 1	528.77 + 175.64	1148.41 + 202.50	
Visit 2	506.32 + 167.98	1100.46 + 228.49	

## Discussion

The purpose of this study was to determine the impact of an Active Nutrition Program on weight loss and metabolic health in overweight and obese adults, compared to usual care. In accordance with our hypotheses, participating in the Active Nutrition program for 60 days resulted in significant weight loss of approximately 6.5 pounds, with a 2-pound weight gain noted for subjects in the usual care group. Related to this, reductions were noted in BMI, as well as waist (4 cm) and hip (3 cm) circumference for the Active Nutrition group. These changes were not noted in the usual care group, emphasizing the need for a weight loss intervention. Our findings are not surprising and are similar to results found in prior studies following the adoption of a weight loss program that limits the number of calories with the incorporation of a semi-structured plan that includes whole foods [8]. As such, these results show the importance and reliability of such strategies and reinforce the need for caloric moderation and mindful food choices.

In addition, clinically relevant changes were noted for reductions in blood pressure (5 mm Hg) and triglycerides (12 mg/dL) for the Active Nutrition group. These findings highlight the broader health benefits of the program, as hypertension is a critical risk factor for cardiovascular disease [2,11]. There are also promising results for the impact the Active Nutrition program has on insulin levels, where the Active Nutrition group averaged a fasting insulin level of 18.64 µU/mL at 60 days post-intervention compared to a fasting insulin level of 28.68 µU/mL for the control group (35% reduction). With the normal range of fasting insulin levels being < 25 µU/mL, the outcomes suggest the potential for the Active Nutrition program to improve insulin levels to within a normal range [29]. The advantages of a diet that supports optimal insulin levels include improved blood sugar control, enhanced metabolism, reduced risk of type 2 diabetes, better weight management, and overall support of long-term health [29,30].

Apart from observing improvements in body composition and metabolic factors, this study also identified positive results for participants’ subjective feelings of well-being. Participants in the Active Nutrition group reported significantly higher average scores in their self-esteem, motivation, endurance, physical appearance, and strength. These findings align with other studies that show that successful and rapid weight loss can positively impact individuals’ mental well-being and motivation to adhere to a program [19-22]. Finally, the above was achieved without subjects reporting any significant increase in hunger, with values remaining relatively consistent across time for both Active Nutrition and usual care subjects. As expected, the total caloric intake decreased over time for subjects in the Active Nutrition group (~450 calories/day), while remaining relatively stable for subjects in the usual care group.

Despite the insights provided by this research, study limitations must be noted. First, a limitation of the study design was the relatively small sample size of 60 participants, which could limit the generalizability of the findings. Along these lines, the absence of diversity in the study sample, predominantly comprising women, limits the application of the results to a more heterogeneous demographic. Although the sample population was not diverse, it also shows the potential health benefits that overweight and obese individuals with comorbidities could have from this program. Lastly, the study’s duration of 60 days, while sufficient to capture short-term effects, may not address the long-term sustainability and maintenance of the weight loss. It is important to note that this study encouraged a balanced and sustainable approach to eating by emphasizing the importance of consuming nutrient-dense foods, which can help individuals meet their nutritional needs while managing caloric intake.

## Conclusions

The Active Nutrition dietary program demonstrated efficacy in inducing weight loss, improving body composition, and positively influencing metabolic levels for men and women seeking weight loss. In addition, Active Nutrition participants had strong adherence to the program and experienced significant improvements in several perceived psychological factors related to overall well-being. Follow-up studies including longer-term designs and larger sample sizes are warranted to determine the sustainability and generalizability of this nutrition program on weight loss and markers of metabolic health. Such studies could include a more holistic approach that not only includes balanced nutrition and mindful eating but also involves regular physical activity and effective lifestyle behavioral strategies to impact change.
